# Caerin 1.1/1.9 peptides control *Acinetobacter baumannii* infection through combined antibacterial and host-directed immunomodulatory mechanisms

**DOI:** 10.3389/fmicb.2026.1777814

**Published:** 2026-05-14

**Authors:** Hongyin Wu, Junjie Li, Jinyi Wu, Quanlan Fu, Yuandong Luo, Yongxin Liang, Furong Zhong, Bin Xu, Zhijun Lin, Tianfang Wang, Xiaosong Liu, Guoying Ni

**Affiliations:** 1 The First Affiliated Hospital, School of Clinical Medicine, Guangdong Pharmaceutical University, Guangzhou, China; 2 Zhongao Biomedical Technology (Guangdong) Co., Ltd., Zhongshan, China; 3 Medical College, Guizhou University, Guiyang, China; 4 Centre for Bioinnovation, University of the Sunshine Coast, Maroochydore, QLD, Australia; 5 School of Science, Technology and Engineering, University of the Sunshine Coast, Maroochydore, QLD, Australia

**Keywords:** *Acinetobacter baumannii*, antimicrobial peptides, immune regulation, immunomodulation, macrophage polarization

## Abstract

**Introduction:**

*Acinetobacter baumannii* is a major multidrug-resistant (MDR) pathogen associated with high morbidity and mortality, driven not only by antibiotic resistance but also by its ability to persist intracellularly and disrupt host immune homeostasis. Host-directed antimicrobial strategies that combine bactericidal activity with immune modulation are therefore urgently needed. Caerin peptides 1.1 (F1) and 1.9 (F3), derived from Australian tree frogs, have demonstrated broad antimicrobial activity, but their combined antibacterial and immunomodulatory mechanisms remain incompletely understood.

**Methods:**

We evaluated the antimicrobial and host-directed effects of F1/F3 using *in vitro* A549 epithelial infection models and an in vivo murine skin-injury infection model. Antibacterial activity was assessed by minimum inhibitory concentration (MIC) assays and colony-forming unit (CFU) quantification. Host cell responses were analyzed using viability, LDH release, reactive oxygen species (ROS), and apoptosis assays. Immune modulation in vivo was investigated by flow cytometry, multiplex immunofluorescence, and cytokine profiling.

**Results:**

F1/F3 exhibited potent antibacterial activity against *A. baumannii* (MIC 3.75 μg/mL) with minimal cytotoxicity to host cells. The peptides significantly reduced intracellular bacterial burden while alleviating infection-induced oxidative stress and early apoptosis. In vivo, F1/F3 treatment reduced bacterial colonization, improved tissue integrity, and reshaped the immune microenvironment. This included increased macrophage infiltration with a shift toward a reparative phenotype, reduced neutrophil accumulation, enhanced dendritic cell presence, and coordinated upregulation of pro- and anti-inflammatory cytokines, including IL-6, IL-12, IFN-γ, IL-10, and TGF-β, without altering TNF-α levels.

**Discussion:**

These findings demonstrate that F1/F3 exert a dual mode of action by combining direct antibacterial effects with host-directed immunomodulation. By integrating microbial suppression, immune reprogramming, and tissue repair, caerin peptides represent promising candidates for the development of next-generation therapies against MDR *A. baumannii* infections.

## 1 Introduction

*Acinetobacter baumannii* has emerged as one of the most formidable nosocomial pathogens worldwide, posing a severe risk, particularly to critically ill and immunocompromised patients (Chen et al., 2025; Luo et al., 2025; Satlin et al., 2025). Its remarkable environmental adaptability, rapid genetic evolution, and extensive antimicrobial resistance have rendered its control increasingly difficult under conventional antibiotic therapy (Marino et al., 2024; Mea et al., 2021). Global surveillance data indicate that nearly half of *A. baumannii* infections are multidrug-resistant (MDR), with resistance rates exceeding 70% in regions such as Latin America and the Middle East, substantially higher than those of most other Gram-negative bacteria (Jamshed and Danaf, 2026). Reflecting the clinical urgency posed by this pathogen, the World Health Organization (WHO) has classified carbapenem-resistant *A. baumannii* (CRAB) as a critical-priority pathogen in the Bacterial Pathogen Priority List (BPPL) (Satlin et al., 2025). The widespread overuse and misuse of antibiotics have accelerated the global antimicrobial resistance (AMR) crisis, progressively eroding the effectiveness of existing antibacterial therapies (Antimicrobial Resistance Collaborators, 2022; Nammi et al., 2025; Nardulli et al., 2022). Despite the stagnation in novel antibiotic development over the past decades, the burden of *Acinetobacter baumannii* infections has continued to rise. Multicenter studies conducted between 2020 and 2025 consistently report high mortality rates in patients with *A. baumannii* bacteremia: 30-day mortality reached 28–56% for CRAB infections (Alwazzeh et al., 2025; Shi et al., 2024), while even antibiotic-susceptible strains are associated with mortality rates of 14–18% (Alwazzeh et al., 2025). Resistance to last-line agents continues to rise, with meropenem resistance exceeding 90% and colistin resistance rising from 1.8 to 8.9% (Castanheira et al., 2023). The urgent need for alternative therapeutic strategies is emphasized by the narrowing therapeutic window of conventional antibiotics. Moreover, appropriate antimicrobial regimens are achievable in fewer than 40% of cases, and colistin treatment is frequently complicated by nephrotoxicity, affecting to 42% of patients (Alwazzeh et al., 2025). Collectively, these data highlight a narrowing therapeutic window for conventional antibiotics and the urgent need for alternative strategies to combat *A. baumannii* infection.

In this context, host-defense peptides (HDPs) have attracted growing interest as promising candidates for next-generation anti-infective therapies (Cavallazzi Sebold et al., 2023; Hetta et al., 2024; Oliveira Júnior et al., 2025; Shriwastav et al., 2025). Beyond direct bactericidal activity, many HDPs exhibit immunomodulatory properties that allow them to modulate host immune responses, enhance pathogen clearance, and promote tissue repair. Such dual antimicrobial and host-directed activities make AMPs particularly attractive for treating infections in which pathogen virulence and host immune dysregulation jointly contribute to disease severity. Among these, the naturally occurring peptides F1 and F3, originally isolated from Australian tree frogs, have demonstrated broad antibacterial, antiviral, and antitumor activities (Fu et al., 2024b; Liu et al., 2025). F1 displays cytotoxic activity against multiple cancer cell types (Fu et al., 2024b), whereas F3 exhibits potent antibacterial effects against a wide range of Gram-positive and Gram-negative bacteria, including *A. baumannii*, *Staphylococcus aureus* (including MRSA), pathogens for which HDP-based approaches hold particular promise given their dual antimicrobial and antibiofilm activities (Chen et al., 2021; Zhang et al., 2023). These peptides are heat-stable and maintain structural integrity across physiologically relevant pH conditions (5.5–7.4), enhancing their translational potential (Yang et al., 2022). Mechanistically, they are proposed to act via a carpet-like model, whereby peptide aggregation on bacterial membranes disrupts membrane integrity and leads to cell death (Chen et al., 2021). Notably, F3 retains activity against resistant bacterial strains and does not readily induce resistance after repeated *in vitro* exposure (Chen et al., 2021). Previous studies have also suggested that combining F1 and F3 may enhance antimicrobial efficacy. For example, a temperature-sensitive gel containing both peptides effectively inhibited MRSA in a skin infection model, and peptide-functionalized magnesium alloy surfaces suppressed MRSA growth *in vitro*, highlighting potential applications in wound treatment and biomedical implants (Chen et al., 2021; Wang T. et al., 2021). Despite these advances, the mechanisms by which caerin peptide combination controls infection, particularly in the context of host immune modulation, remain incompletely understood.

While the ultimate clinical target of host-directed therapies is MDR *A. baumannii* infections, we used the well-characterized *A. baumannii* reference strain GDM1.609 to initially dissect the antibacterial and immunomodulatory mechanisms of the F1/F3 combination. This approach minimizes confounding variables and provides a solid mechanistic framework, which can be validated with MDR isolates in future studies. In this study, we investigated the antibacterial and immunomodulatory functions of F1/F3 in the context of *A. baumannii* infection. We demonstrate that F1/F3 not only directly suppresses bacterial *in vitro* but also reshapes the host immune microenvironment, promoting coordinated bacterial clearance and tissue repair *in vivo*. These findings provide mechanistic insight into the dual antibacterial and host-directed activities of caerin peptides and establish a foundation for their further optimization and clinical translation as host-directed therapeutics against MDR infections.

## 2 Materials and methods

### 2.1 Animals

All animal experiments were approved by the Ethics Committee of Animal Experiments at the First Affiliated Hospital of Guangdong Pharmaceutical University (approval No.: GYFYG2R202326) and conducted in accordance with ethical standards. Female BALB/c mice (6–8 weeks old) were obtained from the Guangdong Province Animal Resource Center (Guangzhou, China). Mice were maintained under specific pathogen-free (SPF) conditions at 22 °C with 60% humidity for a 12 h light/dark cycle, with sterile food and water provided ad libitum. After the completion of the animal experiment cycle, mice were anesthetized with an intraperitoneal injection of 50 mg/kg sodium pentobarbital, followed by cervical dislocation.

### 2.2 Cell culture

Human A549 cells were provided by the Experimental Center of the First Affiliated Hospital of Guangdong Pharmaceutical University. Cells were cultured in complete DMEM (Gibco) containing 10% heat-inactivated fetal bovine serum (FBS; Gibco), 100 U/mL penicillin, and 100 μg/mL streptomycin at 37 °C with 5% CO₂.

### 2.3 Bacterial strains

The *A. baumannii* strain (GDM1.609) was purchased from the Guangdong Microbial Culture Collection Center. Revival and storage procedures were performed as previously described (Chen et al., 2021). Routine bacterial culture was performed in Mueller–Hinton (MH) broth or on agar plates at 37 °C. During A549 cell infection experiments, cultures were maintained under 5% CO₂ to match the host cell culture conditions, rather than because *A. baumannii* requires CO₂ supplementation for growth.

### 2.4 Peptide synthesis

Caerin 1.1 (F1, GLLSVLGSVAKHVLPHVVPVIAEHL-NH₂) and caerin 1.9 (F3, GLFGVLGSIAKHVLPHVVPVIAEKL-NH₂) were synthesized by Mimotopes Proprietary Limited (Wuxi, China). All peptides had >99% purity and endotoxin levels <0.44 EU/mL. F1 and F3 were reconstituted as described previously (Chen et al., 2021). Briefly, F1 and F3 were each dissolved in sterile PBS to prepare 1 mg/mL stock solutions, aliquoted, and stored at −20 °C until use. Before each experiment, the stock solutions were diluted to the required working concentrations. For combination treatment, equal volumes of F1 and F3 stock solutions were mixed and diluted to the desired final concentrations at a 1:1 ratio (μg/mL), as previously described (Antimicrobial Resistance Collaborators, 2022). The F1/F3 combination showed synergistic activity, with a fractional inhibitory concentration index (FICI) of <0.5 (Chen et al., 2021).

### 2.5 MIC determination

Minimal inhibitory concentrations (MICs) of F1/F3 (the mixture of F1 and F3 at a 1:1 ratio) against *A. baumannii* were determined using the broth microdilution assay following CLSI guidelines (Hong et al., 2026). Bacterial suspensions were adjusted to an OD_590_ of 0.09–0.10, diluted 1:100, and seeded at 100 μL per well in 96-well plates. F1/F3 were serially diluted in MH to 15, 7.5, 3.75, 1.875, 0.9375, 0.46875, and 0.2 μg/mL, respectively. After initial two-fold serial dilution screening, an intermediate concentration (2 μg/mL) was included to refine MIC determination within the predicted inhibitory range. Mueller–Hinton broth was added as the growth control. Plates were incubated overnight at 37 °C, and OD₅₉₀ values were measured using a MULTISKAN GO microplate reader, which provides equivalent assessment of bacterial growth to OD_600_ in this detection system. Specifically, the MICs or treatment concentrations of F1/F3 described herein refer to the respective final concentrations of each individual peptide within the mixed system. Therefore, an MIC of 3.75 μg/mL for F1/F3, as stated in this text, indicates that the final concentrations of both F1 and F3 are 3.75 μg/mL; this definition is consistent with the terminology used in our previously published articles.

### 2.6 MTT assay

A549 cells (1 × 10^4^ cells/well) were seeded in 96-well plates and incubated for 24 h. Cells were treated with F1/F3 (0–20 μg/mL) for 18 h, followed by incubation with 10 μL MTT reagent (Beyotime) for 4 h. Formazan crystals were dissolved in 50 μL DMSO (Sigma-Aldrich, St. Louis, MO, USA), and absorbance at 540 nm was measured. Control wells (untreated cells) were processed identically and served as the reference for calculating cell viability.

### 2.7 *In vitro* infection models

#### 2.7.1 Construction of A549 infection model

A549 cells (2 × 10^5^ cells/well) were seeded in 24-well plates. *A. baumannii* cultures grown overnight in MH broth were adjusted to an OD₆₀₀ of 0.09–0.10, pelleted (2,780 g, 10 min, 4 °C), and resuspended in serum-free DMEM. Cells were washed three times with PBS and infected at a multiplicity of infection (MOI) of 50. Plates were centrifuged at 200 × g to facilitate bacterial contact and then incubated at 37 °C under 5% CO₂ for 24 h to maintain the A549 cell culture environment.

#### 2.7.2 Extracellular bacterial burden assay

After infection, cells were incubated in 1 mL DMEM containing either 1 × MIC (3.75 μg/mL) F1/F3 or vehicle for 6 h. Culture supernatants were then collected, serially diluted, and plated on agar for CFU enumeration. Because unbound bacteria were not removed before supernatant collection and host cell-associated bacteria were not separately recovered in this assay, the readout represents extracellular bacterial burden in the culture supernatant rather than a specific adhesion index. After 24 h of incubation, the colonies were counted.

#### 2.7.3 Intracellular infection assay

At 24 h post-infection, cells were washed three times and incubated with PBS containing 100 μg/mL Gentamicin for 1 h to eliminate extracellular bacteria. Cells were washed again and incubated with DMEM ± 1 × MIC (3.75 μg/mL) F1/F3 for 6 h. Cells were lysed in 1% Triton X-100 for 10 min, and intracellular bacteria were serially diluted and spot-plated onto agar plates. After 24 h of incubation, the colonies were counted.

### 2.8 Pretreatment of *A. baumannii* with F1/F3

Bacteria were pretreated with 1 × MIC (3.75 μg/mL) F1/F3 for 6 h prior to infection. Infection and intracellular CFU quantification were performed as described above.

### 2.9 Lactate dehydrogenase (LDH) release assay

A549 cells (1 × 10^4^ cells/well) were seeded into 96-well plates and infected with *A. baumannii* (MOI 50) for 24 h. Supernatants were collected, and LDH activity was quantified using an LDH assay kit (Beyotime) following the manufacturer’s protocol.

### 2.10 Measurement of reactive oxygen species

A549 cells (2 × 10^5^ cells/well) were seeded into 24-well plates and infected with *A. baumannii* (MOI 50) for 24 h. Intracellular ROS levels were detected using DCFH-DA (Beyotime) (He et al., 2024). A549, A549 + *A. baumannii*, and A549 + *A. baumannii* + F1/F3 groups were incubated for 24 h, washed, and incubated with DCFH-DA for 30 min. Fluorescence images were acquired using a fluorescence microscope (Nikon), and signal intensity was quantified with ImageJ.(20 × objective excitation 488 nm, emission 525 nm).

### 2.11 Apoptosis analysis

Cell apoptosis was assessed using an Annexin V-FITC/PI kit (Beyotime). A549 cells (5 × 10^5^/well) were infected with F1/F3-pretreated *A. baumannii* (MOI 50) for 24 h. Cells were trypsinized (EDTA-free), stained with 5 μL Annexin V-FITC and 10 μL PI for 10–20 min in the dark, and analyzed by flow cytometry.

### 2.12 ELISA

Supernatants collected from A549 cells infected with untreated or F1/F3-pretreated *A. baumannii* (MOI 50) for 6 or 24 h were assayed for TNF-*α* and IL-6 using ELISA kits (BioLegend, San Diego, CA, USA). Briefly, 100 μL of each standard or sample was added to antibody-coated 96-well plates and incubated for 2 h at room temperature. After washing, plates were incubated with biotinylated detection antibody for 1 h, followed by avidin-HRP for 30 min. Tetramethylbenzidine (TMB) substrate was added for 15 min, and the reaction was stopped with 2 N H₂SO₄. Absorbance was measured at 450 and 570 nm (for wavelength correction) using a MULTISKAN GO microplate reader. Cytokine concentrations were calculated by interpolation from standard curves generated with recombinant cytokines provided in the kits. The detection limits were 4 pg./mL for TNF-*α* and 2 pg./mL for IL-6.

### 2.13 Mouse skin infection model

BALB/c mice (6–8 weeks old) were anesthetized with an intraperitoneal injection of 50 mg/kg sodium pentobarbital, using a 1 mL syringe (KDL, Shanghai). Dorsal hair was shaved, and a 5-mm full-thickness skin wound was generated using a biopsy punch (TexBio, Guangzhou). *A. baumannii* in logarithmic phase was applied onto wounds at 1 × 10^8^ CFU (10 μL). Mice received PBS or F1/F3 (100 μL, 10 μg/mL) daily for 2 days starting 1-day post-infection, which was gently applied dropwise onto the wound surface using a micropipette, ensuring complete coverage of the wound. After anesthetizing the mice with an intraperitoneal injection of 50 mg/kg sodium pentobarbital, cervical dislocation was performed. Skin tissues (~1 cm × 1 cm) were homogenized using a sterile glass homogenizer (5 mL, Beyotime), and the bacterial suspension was serially diluted and spot-plated onto agar plates. After 24 h of incubation, the colonies were counted.

### 2.14 Histology (H&E staining)

Wound tissue sections were first dewaxed with xylene I and xylene II (15 min each), then rehydrated with a gradient of ethanol solutions: 100% ethanol I and II (5 min each), followed by 95, 90, 80, 70, and 50% ethanol (5 min each). The sections were then rinsed three times with distilled deionized water (ddH2O). Nuclear staining was performed using Harris hematoxylin (BASO, Zhuhai) for 20 min, followed by rinsing with tap water for 30 min to achieve bluing, taking care to avoid direct water flow. After rinsing three times with ddH2O, the sections were dehydrated with 50, 70, 80, and 90% ethanol (2 min each). Cytoplasmic counterstaining was performed using eosin Y solution (BASO, Zhuhai) for 10 s, followed by dehydration with 95% ethanol I and II (2 min each), then 95% ethanol II (2 min), 100% ethanol I (5 min), and 100% ethanol II (5 min), and finally cleared with xylene I and xylene II (5 min each). Slides were permanently mounted with neutral resin and examined under a ZEISS AX10 light microscope for image acquisition.

### 2.15 Flow cytometry

Wound tissues (~1 cm^2^) were processed into single-cell suspensions and stained with fluorophore-conjugated antibodies (Table 1). Data was acquired on a BD FACS Aria II cytometer and analyzed using FlowJo software. In flow cytometry, Ly6C gating was employed as a preliminary screening method to distinguish inflammatory macrophages (Ly6C^+^) from tissue-resident macrophages (Ly6C^−^).

**Table 1:** Flow cytometry antibodies used in this study.

Antibody	Label	Article number	Clone	Provider
CD45.2	APC	17-0042-83	RM4-5	eBioscience
CD62L	FITC	11-0621-81	MEL-14	eBioscience
CD45.2	FITC	11-0454-85	104	eBioscience
CD11b	Percp-cy5.5	45-0112-82	M1/70	eBioscience
Ly-6G	BV421	108445	RB6-8C5	BioLegend
Ly6C	BV421	562,727	AL-21	BD Bioscience
CD11c	PE-Cy7	558,079	HL3	BD Bioscience
CD8a	PE-Cy7	552,877	53–6.7	BD Bioscience
MHC-II	PE	12-5322-81	NIMR-4	eBioscience
F4/80	PE	12-4801-82	BM8	eBioscience
CD3e	APC-cy7	557,596	145-2C11	BD Bioscience
CD4	APC	100,412	GK1.5	BioLegend

### 2.16 RNA extraction, cDNA synthesis, and qPCR

Skin tissues were collected and stored at −80 °C. Total RNA was extracted using the Animal Tissue RNA Kit (Goonie, Guangzhou) with additional homogenization using 3-mm sterile zirconium beads (Servicebio). cDNA was synthesized using HiScript® IV All-in-One Ultra RT SuperMix (Vazyme). qPCR was performed on a Light Cycler 480 system (Roche). Relative gene expression was calculated using the 2^−^ΔΔCt method with GAPDH as the reference gene. The PBS control group was set as the calibrator (value = 1) for each target gene. Primer sequences are listed in Table 2.

**Table 2:** The sequence information of each primer used for mouse gene expression analysis.

Name	Sequence
GAPDH-F	GGCCTCCAAGGAGTAAGAAA
GAPDH-R	GCCCCTCCTGTTATTATGG
TNF-α-F	TCTACTCCCAGGTTCTCTTCA
TNF-α-R	CCTGGTATGAGATAGCAAATCG
IL-6-F	GTTGCCTTCTTGGGACTGAT
IL-6-R	TTTCCACGATTTCCCAGAGA
IL-1β-F	TCTCGCAGCAGCACATCAACA
IL-1β-R	AATGGGAACGTCACACACCAGC
TGF-β-F	GGACCCTGCCCCTATATTTG
TGF-β-R	AGGAGCGCACAATCATGTTG
IFN-γ-F	ATGGCTGTTTCTGGCTGTT
IFN-γ-R	TCCTTTTGCCAGTTCCTCCA
IL-12-F	ACGAGAGTTGCCTGGCTACTAG
IL-12-R	CCTCATAGTGCTACCAAGGCAC
IL-10-F	AAGCCTTATCGGAAATGATCCA
IL-10-R	GCTCCACTGCCTTGCTCTTATT

### 2.17 Multiplex immunofluorescence staining

Multiplex immunofluorescence staining was performed on formalin-fixed paraffin-embedded skin sections following a previously described tyramide signal amplification (TSA)-based protocol (Stack et al., 2014). Briefly, after dewaxing and rehydration, antigen retrieval was performed in EDTA buffer (pH 9.0) using a pressure cooker. Endogenous peroxidase was blocked with 3% H₂O₂, followed by blocking with 10% goat serum. Sequential staining was performed for Ly6G, CD86, F4/80, and CD206 using primary antibodies (Table 3) and HRP-conjugated secondary antibodies, with TSA amplification using iFluor® tyramide conjugates. Between staining cycles, antibodies were stripped using low-pH citrate buffer. Nuclei were counterstained with DAPI, and slides were mounted with antifade mounting medium (Vector Laboratories). Images were acquired using a Pannoramic SCAN II whole-slide scanner (3DHistech) and analyzed with CaseViewer software.

**Table 3:** Antibodies used for multiplex immunofluorescence staining.

Target	Host species	Vendor	Catalog number	Dilution
Ly6G	Rabbit	Abcam	ab238132	1:5000
CD86	Rabbit	CST	19,589	1:400
F4/80	Rabbit	CST	70,076	1:4000
CD206	Rabbit	CST	24,595	1:2000
Secondary Antibody (HRP)	Goat Anti-Rabbit	Abcam	ab205718	1:4000

CD86 (M1 macrophage marker) and CD206 (M2 macrophage marker) immunofluorescence staining were also employed as validation methods to confirm the functional phenotypes of M1 and M2 macrophages. The labeling and quantitative analysis of CD86 and CD206 enabled precise validation of the functional characteristics of macrophage subsets identified by Ly6C gating, elucidating their polarization status under different treatments.

### 2.18 Statistical analysis

Flow cytometry data were analyzed using Flow Jo v10. Results are presented as mean ± SD. Statistical analyses were performed using GraphPad Prism 9. Group comparisons were conducted using Student’s *t*-test or one-way ANOVA as appropriate.

## 3 Results

### 3.1 *In vitro* susceptibility of *A. baumannii* and A549 cells to F1/F3

**Figure 1:**
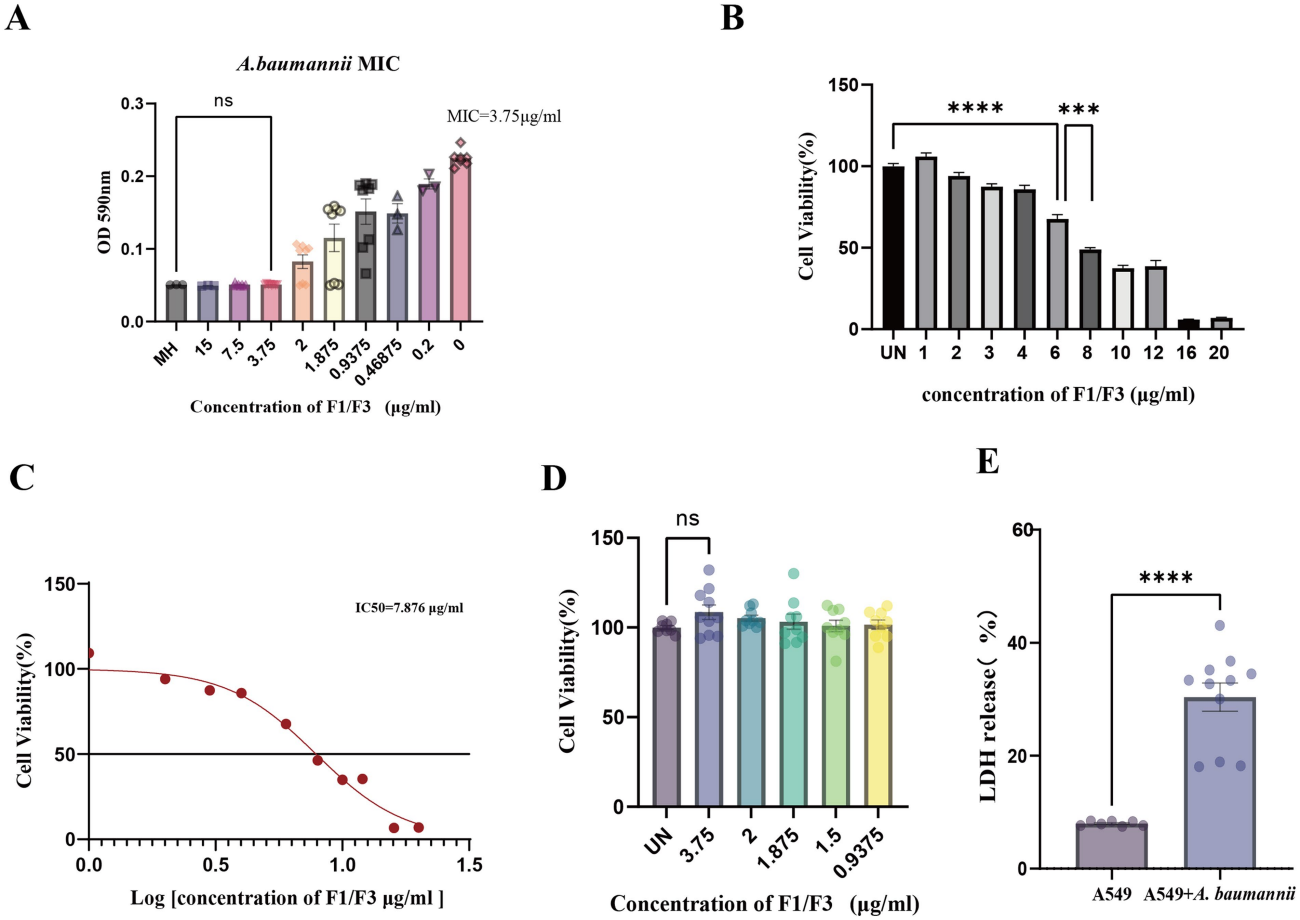
*In vitro* effects of F1/F3 on *A. baumannii* and A549 cells. **(A)** Minimum inhibitory concentration of F1/F3 against *A. baumannii*, showing complete growth inhibition at 3.75 μg/mL. **(B)** Dose-dependent changes in A549 cell viability following treatment with F1/F3 (1–20 μg/mL), as determined by the MTT assay. **(C)** IC₅₀ value of F1/F3 in A549 cells (7.876 μg/mL), as determined by the MTT assay. **(D)** Effect of F1/F3 on A549 cells at 1 MIC (3.75 μg/mL), as determined by the MTT assay. **(E)** LDH release in A549 cells following infection with *A. baumannii* for 24 h at MOI = 50. Error bars indicate the mean ± SD from three independent experiments (*n* = 3). Statistical significance was defined as ns, not significant; ***p < 0.001; ****p < 0.0001 (unpaired *t*-test for **A,B,D,E)**.

We first examined the antibacterial efficacy of F1/F3 against *A. baumannii*. MIC testing showed that F1/F3 markedly inhibited bacterial growth, yielding a MIC value of 3.75 μg/mL (Figure 1A). To evaluate host-cell susceptibility, A549 epithelial cells were exposed to escalating concentrations of F1/F3 (1–20 μg/mL). F1/F3 reduced A549 cell viability in a dose-dependent reduction (Figure 1B), with an IC₅₀ of 7.876 μg/mL as determined by MTT assay (Figure 1C). Notably, at the MIC concentration (3.75 μg/mL), F1/F3 did not significantly affect A549 cell viability or induce LDH release, indicating minimal cytotoxicity under these conditions, as cell viability (OD_540_) remained comparable to that of the untreated group (Figure 1D). These findings indicated that F1/F3 is selectively antimicrobial and does not compromise host-cell viability within its effective bactericidal range. To quantify host-cell damage caused by bacterial infection, LDH release was measured following exposure of A549 cells to *A. baumannii* (MOI = 50) for 24 h. LDH levels increased significantly, reaching 18.4% (Figure 1E), confirming substantial membrane damage and cell death induced by *A. baumannii* infection.

### 3.2 F1/F3 suppresses intracellular *A. baumannii* infection

**Figure 2:**
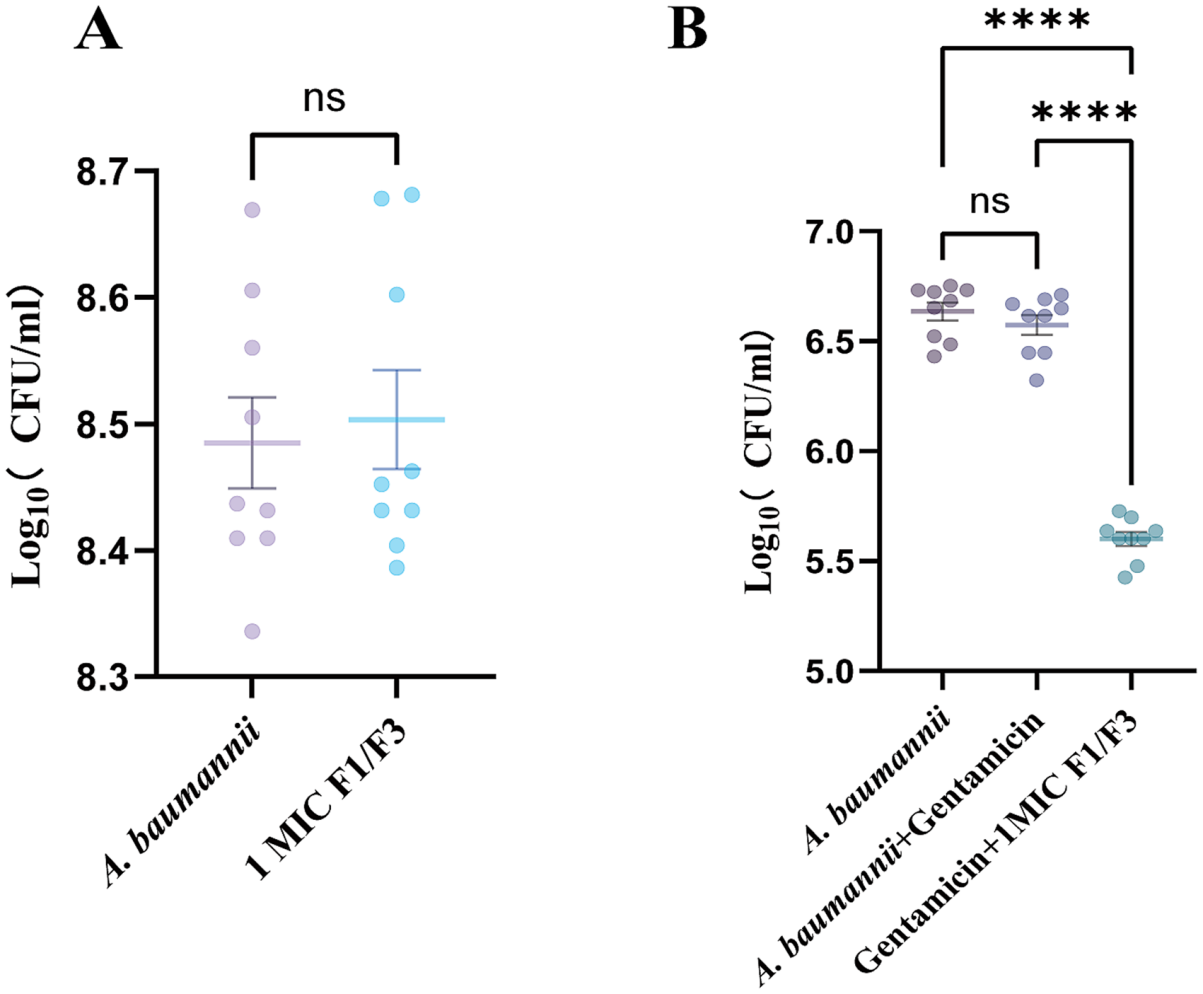
F1/F3 suppresses intracellular *A. baumannii* in A549 cells. **(A)** CFU counts in culture supernatants after 6 h of F1/F3 treatment. **(B)** Intracellular CFU following gentamicin treatment to remove extracellular bacteria, followed by 6 h of F1/F3 exposure. Error bars indicate the mean ± SD from three independent experiments (*n* = 3). Statistical significance was defined as ns, not significant; ****p < 0.0001 [unpaired *t*-test for **(A)**; one-way ANOVA for **(B)**].

To assess the intracellular antimicrobial activity of F1/F3, an A549 epithelial cell infection model was established. Following infection with *A. baumannii* at MOI = 50 for 24 h, cells were treated with F1/F3 at 1 × MIC (3.75 μg/mL) for 6 h. CFU enumeration from culture supernatants revealed no significant difference between untreated and F1/F3-treated cultures (8.39 log vs. 8.41 log), indicating that under the host-cell infection conditions, the peptide treatment did not measurably reduce extracellular bacterial counts (Figure 2A and Supplementary Figure S1A). Therefore, any potential effect of F1/F3 alone on ROS levels in uninfected cells would be minimal and outside the scope of this study, which focuses on the protective effect of F1/F3 against infection-induced oxidative stress.

To quantify intracellular bacterial burden, extracellular bacteria were eliminated with gentamicin (100 μg/mL for 1 h), after which cells were lysed for CFU recovery. As expected, supernatant CFU remained unchanged following gentamicin and subsequent F1/F3 treatment (Figure 2B). In contrast, intracellular bacterial load was significantly reduced by 0.87 log (6.4 log vs. 5.54 log) following 6 h of F1/F3 treatment compared with the control cells (Figure 2B and Supplementary Figure S1B). Collectively, these results show that while F1/F3 treatment did not significantly reduce extracellular *A. baumannii* under the conditions tested, it markedly decreased the intracellular bacterial burden when assessed after elimination of extracellular bacteria.

### 3.3 F1/F3 pretreatment attenuates *A. baumannii*-induced apoptosis in A549 cells

**Figure 3:**
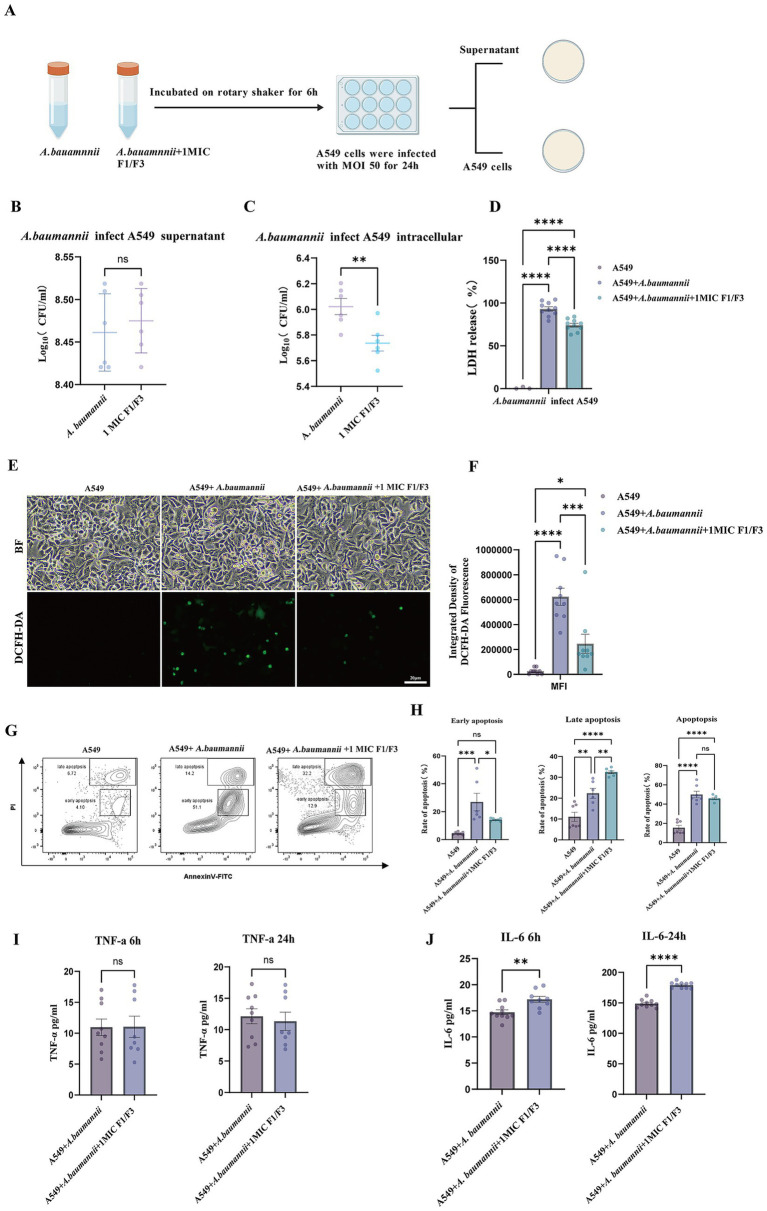
F1/F3 pretreatment reduces intracellular bacterial burden, alleviates cellular injury, and modulates apoptotic and inflammatory responses during *A. baumannii* infection. **(A)** Schematic illustration of the A549 infection model using *A. baumannii* pretreated with F1/F3. Created with Bio GDP (Jiang et al., 2025). **(B)** *A. baumannii* was pretreated with F1/F3 at 1 × MIC (3.75 μg/mL) for 6 h and subsequently used to infect A549 cells at MOI = 50 for 24 h. Culture supernatants were plated on MH agar to quantify extracellular bacterial load. **(C)** Intracellular bacterial burden quantified by plating lysates from infected A549 cells following PBS washes and 0.1% Triton X-100 lysis. **(D)** LDH release in infected A549 cells after 24 h infection with F1/F3-pretreatedor untreated *A. baumannii*. **(E,F)** Intracellular ROS levels in uninfected cells, infected cells, and infected cells exposed to F1/F3, with quantitative analysis shown in **(F)**. **(G)** Flow cytometric analysis of apoptosis under different treatment conditions. **(H)** Quantification of early, late, and total apoptosis across treatment groups. **(I,J)** ELISA quantification of TNF-*α* **(I)** and IL-6 **(J)** secretion at 6 h and 24 h in infected A549 cells with or without F1/F3 pretreatment. Error bars indicate the mean ± SD from three independent experiments (*n* = 3) for panels **(D,F,I,J)**, and from two independent experiments (*n* = 2) for panels **(B,C,G,H)**. ns, not significant; *p < 0.05; **p < 0.01; ***p < 0.001; ****p < 0.0001 [unpaired *t*-test for **(B,C,I,J)**; one-way ANOVA for **(D, F, H)**].

We next examined whether F1/F3 pretreatment modulates *A. baumannii*-induced cytotoxicity and apoptosis in A549 cells. The experimental scheme is illustrated in Figure 3A. Pretreatment of *A. baumannii* with F1/F3 at 1 × MIC (3.75 μg/mL) for 6 h did not alter bacterial load in the culture supernatant (Figure 3B and Supplementary Figure S2A). In contrast, intracellular bacterial burden was significantly reduced, decreasing from 6.15 log to 5.67 log CFU (Figure 3C and Supplementary Figure S2B), indicating that F1/F3 pretreatment impairs the ability of *A. baumannii* to establish intracellular infection. Consistent with reduced bacterial burden, LDH assays showed that F1/F3 pretreatment lowered LDH release to 66% of the infection group (Figure 3D), indicating reduced host-cell damage. Similarly, infection-induced oxidative stress was markedly elevated in infected cells but significantly decreased following F1/F3 pretreatment, as reflected by reduced intracellular ROS levels (Figures 3E,F).

Flow cytometric analysis further revealed distinct effects on apoptosis dynamics. F1/F3 pretreatment significantly reduced early apoptosis from 35.7% in the infection group to 13.9%, whereas late apoptosis increased moderately from 17.9 to 31.6%. As a result, the overall proportion of apoptotic cells did not differ significantly between the infection and F1/F3-pretreated groups (53.6% vs. 45.1%, ns) (Figures 3G,H). Cytokine analysis showed that TNF-*α* secretion remained unchanged at both 6 h and 24 h (Figure 3I), whereas IL-6 production was significantly upregulated at both time points (Figure 3J), indicating a selective regulatory effect of F1/F3 on infection-induced inflammatory responses.

### 3.4 F1/F3 reduces bacterial colonization and alleviates skin inflammation *in vivo*

**Figure 4:**
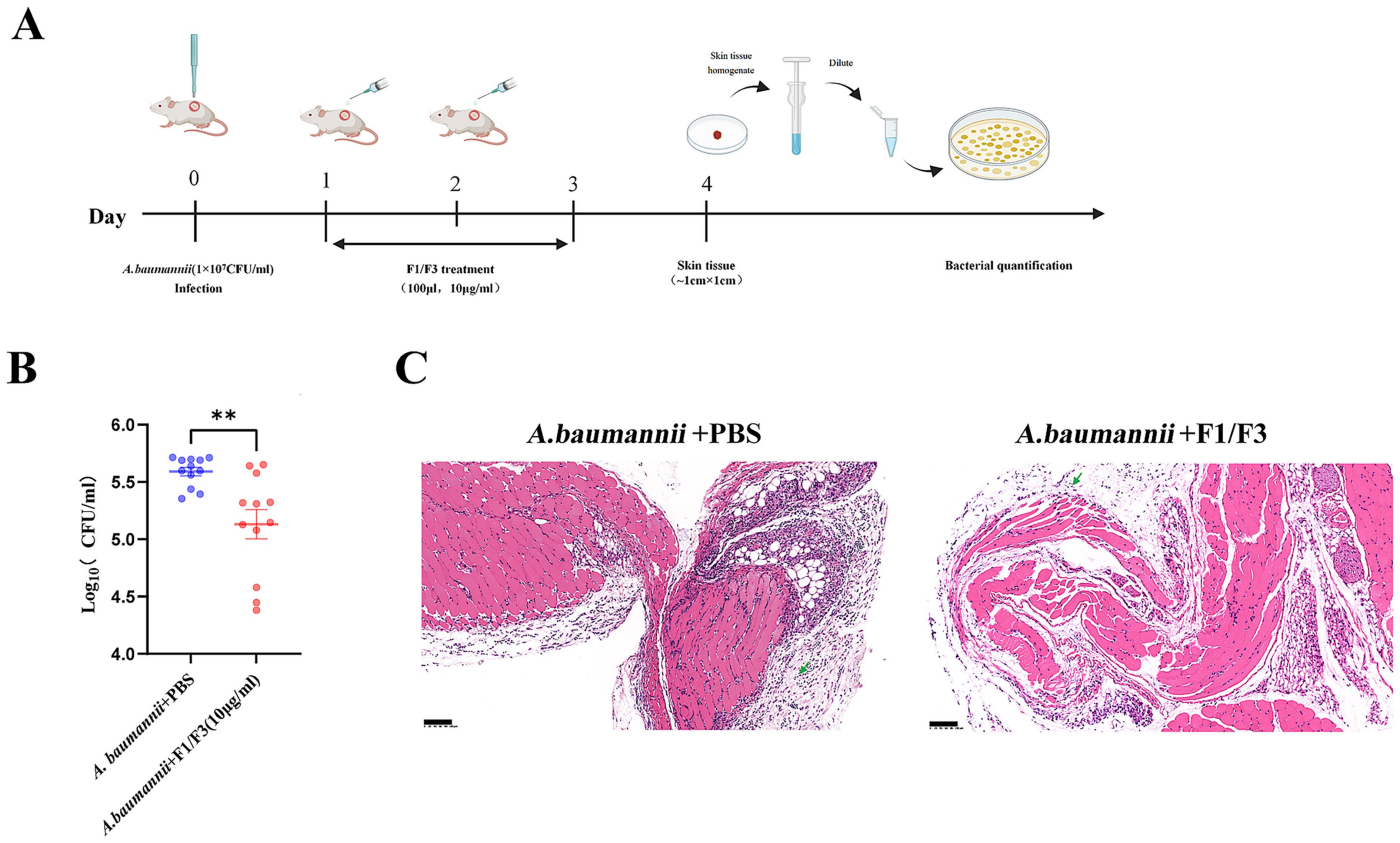
F1/F3 reduces bacterial colonization and skin inflammation in mice. **(A)** Schematic illustration of the mouse skin injury model infected with *A. baumannii*. Created with BioRender.com. **(B)** Bacterial burden in wounded skin tissues from mice infected with *A. baumannii* and treated with PBS or F1/F3 (10 μg/mL), quantified by spot-plating on MH agar. **(C)** Histological analysis of injured muscle tissues collected on day 4 following infection and treatment with PBS or F1/F3 (10 μg/mL), assessed by H&E staining, showed differences in inflammatory infiltration and tissue integrity. Error bars indicate the mean ± SD from two independent experiments (*n* = 2). ns: Not significant; ** *p* < 0.01 [unpaired *t*-test for **(B)**].

To evaluate the therapeutic potential of F1/F3 in vivo, we employed a murine skin-injury infection model (Figure 4A). By day 3, F1/F3 treatment markedly reduced bacterial colonization at wound sites and visibly accelerated wound healing compared with PBS controls (Figure 4B). Quantitative analysis of bacterial burden by CFU plating confirmed significant reductions in bacterial burden in F1/F3-treated wounds (Figure 4B and Supplementary Figure S3A).

Histopathological analysis on day 4 further demonstrated the protective effects of F1/F3. Wound tissue from F1/F3-treated mice exhibited minimal inflammatory cell infiltration and preserved structural integrity, whereas PBS-treated mice showed extensive inflammation and pronounced tissue damage (Figure 4C). Semi-quantitative scoring of inflammation indicated a trend toward lower inflammatory scores in the F1/F3 group, although the difference did not reach statistical significance (Supplementary Figure S3B). Collectively, these findings indicate that F1/F3 effectively suppresses bacterial colonization and mitigates infection-induced inflammation, thereby promoting improved wound healing *in vivo*.

### 3.5 F1/F3 reshapes the immune microenvironment and enhances cytokine responses in *A. baumannii* – infected skin

**Figure 5:**
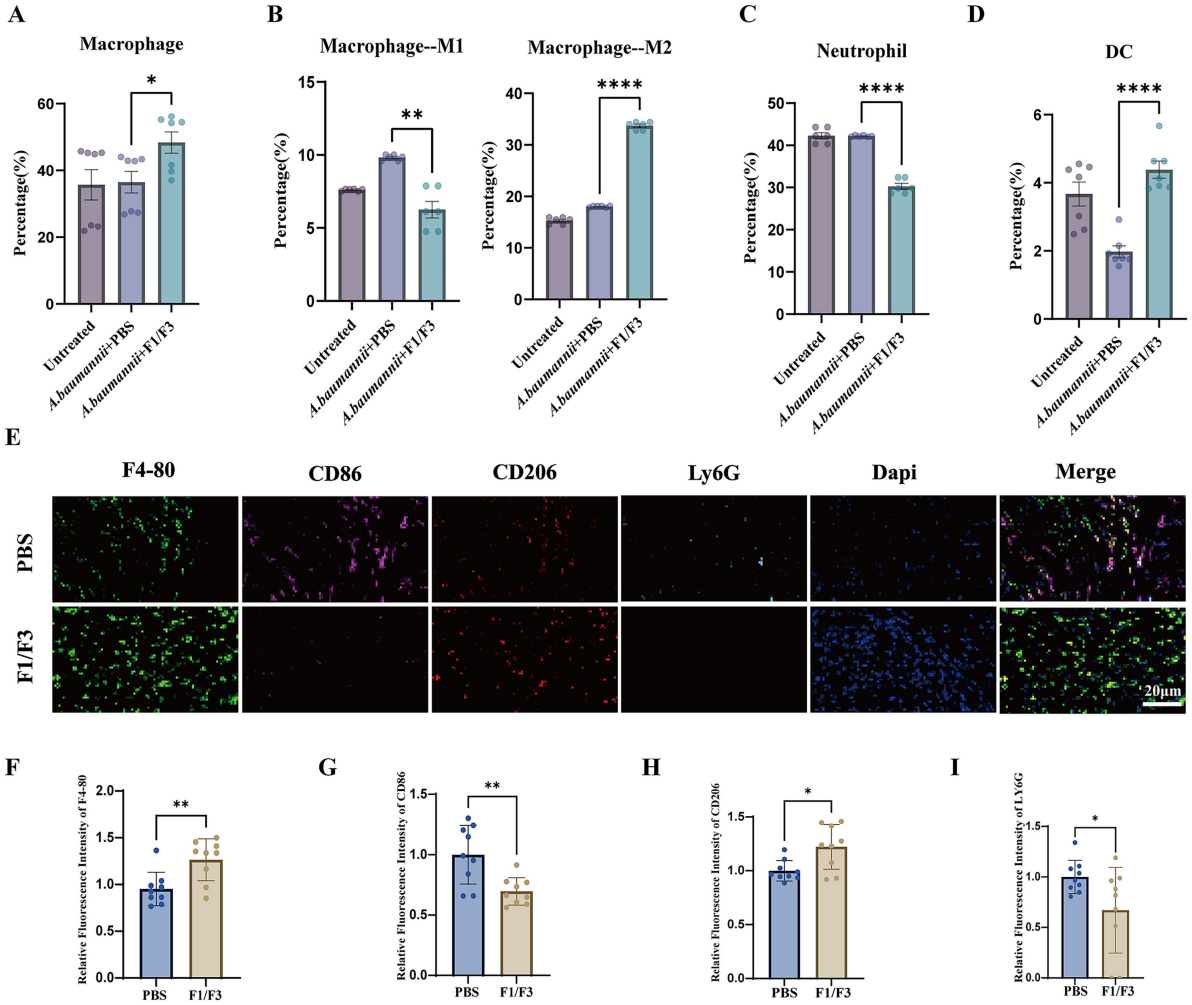
F1/F3 modulates local immune responses during cutaneous *A. baumannii* infection. Flow cytometric analysis of immune cell populations in skin tissues from untreated mice, PBS-treated mice, and F1/F3-treated mice. **(A)** Macrophages identified as CD45^+^CD11b^+^F4/80^+^ cells. **(B)** Macrophage subsets based on Ly6C expression: Ly6C^+^ inflammatory macrophages and Ly6C^−^ tissue-associated macrophages. **(C)** Neutrophils (CD45^+^CD11b^+^Ly6G^+^). **(D)** Dendritic cells (CD45^+^CD11c^+^MHCII^+^). **(E)** Representative multiplex immunofluorescence images of infected skin tissues showing macrophages and neutrophils. **(F–I)** Quantification of macrophage abundance and polarization markers, including CD86^+^ (M1-associated) and CD206^+^ (M2-associated) macrophages, and Ly6G^+^ neutrophils in infected skin tissues. Error bars indicate the mean ± SD from two independent experiments (*n* = 2). ^*^p < 0.05; **p < 0.01; ****p < 0.0001 [unpaired *t-t*est for **(A–D,F–I)**].

Flow cytometry analysis revealed substantial modulation of local immune cell populations following F1/F3 treatment. The proportion of macrophages increased significantly in F1/F3-treated skin compared with PBS controls (39.4% vs. 27.3%) (Figure 5A and Supplementary Figure S5A). Within the macrophage compartment, Ly6C^+^ inflammatory macrophages (M1-like) were significantly reduced in F1/F3-treated mice compared to PBS controls (19.0% vs. 30.1%), while Ly6C^−^ tissue-resident macrophages (M2-like) were markedly expanded (35.7% vs. 12.1%) (Figure 5B and Supplementary Figure S5B). This phenotypic shift was corroborated by multiplex immunofluorescence staining, which showed a decrease in CD86^+^ (M1) macrophages and an increase in CD206^+^ (M2) macrophages following F1/F3 treatment (Figures 5E–I), collectively indicating a reprogramming toward a reparative macrophage phenotype. Neutrophils were substantially decreased in the F1/F3 group (44.3% vs. 70.1%) (Figure 5C and Supplementary Figure S5C), suggesting attenuation of acute inflammatory responses. In contrast, dendritic cells were increased (5.0% vs. 2.3%) (Figure 5D and Supplementary Figure S5D), indicating potential enhancement of local antigen-presentation capacity. T-cell frequencies remained unchanged between groups (Supplementary Figure S4). All representative gate design strategies are shown in Supplementary Figures S6A,B.

**Figure 6:**
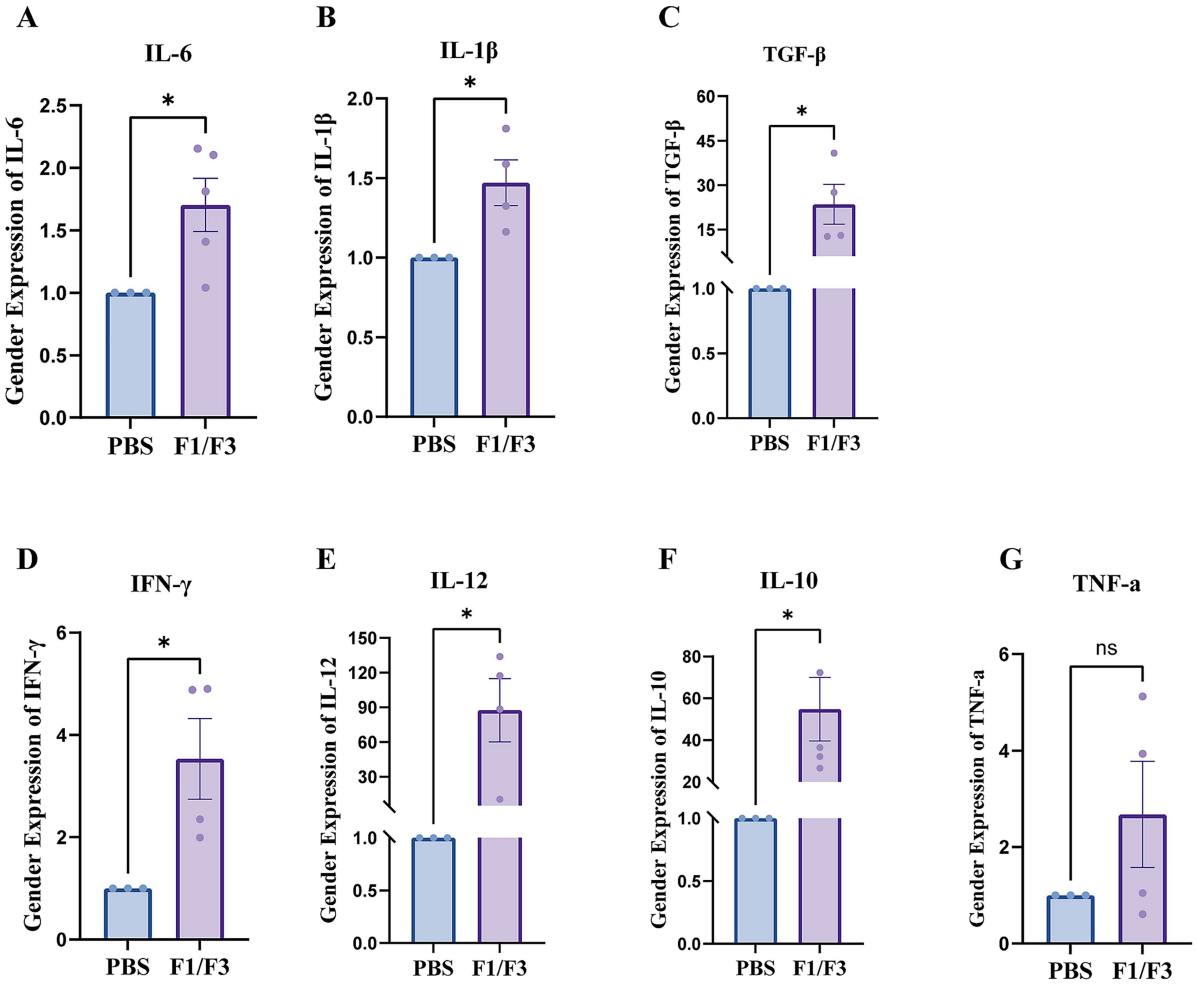
F1/F3 modulates cytokine expression in *A. baumannii*-infected skin. **(A)** IL-6, **(B)** IL-1β, **(C)** TGF-β, **(D)** IFN-γ, **(E)** IL-12, **(F)** IL-10, and **(G)**TNF-α expression levels in skin tissues from *A. baumannii*-infected mice treated with PBS or F1/F3. Cytokine levels were quantified by qPCR. **(A-G)** Relative gene expression was calculated using the 2(^–ΔΔCt^) method, with PBS group normalized to 1. Error bars indicate the mean ± SD from two independent experiments (*n* = 2). ns: not significant; *p < 0.05; [unpaired *t*-test for **(A-G)**].

In parallel, F1/F3 treatment significantly elevated several cytokines involved in inflammatory signaling, adaptive immunity, and tissue repair. Levels of IL-6, IL-1*β*, TGF-β, IFN-*γ*, IL-12, and IL-10 were all markedly increased relative to PBS controls (Figures 6A–F). TNF-*α* showed an increasing trend following F1/F3 treatment, but this change did not reach statistical significance (Figure 6G). Together, these results demonstrate that F1/F3 reshapes the immune microenvironment by reducing inflammatory infiltration and promoting macrophage functional reprogramming, while simultaneously enhancing a broad spectrum of cytokine responses that support immune activation and tissue repair without exacerbating TNF-α–mediated inflammation.

## 4 Discussion

The global rise of MDR bacterial infections, largely driven by the widespread and often inappropriate use of antibiotics, has substantially reduced the effectiveness of many conventional antimicrobial therapies. Among these pathogens, *A. baumannii* has emerged as a major opportunistic pathogen associated with hospital-acquired infections, including ventilator-associated pneumonia, bloodstream infections, and wound infections (Lee et al., 2020; Mukhopadhyay et al., 2025; Tiku et al., 2022; Yang Y. et al., 2024). Clinical isolates of *A. baumannii* frequently exhibit resistance to multiple classes of antibiotics, leading to limited therapeutic options and increased morbidity and mortality. These challenges highlight the urgent need to develop alternative antimicrobial strategies with mechanisms distinct from conventional antibiotics (Moser et al., 2021; Munita and Arias, 2016; Scott et al., 2018). HDPs such as F1 and F3 have attracted increasing attention due to their broad antimicrobial activity and immunomodulatory properties. In this study, we investigated the antibacterial and host-directed effects of the combination F1/F3 against *A. baumannii* using *in vitro* and *in vivo* models, with a particular focus on its ability to modulate host immune responses during infection.

*In vitro*, F1/F3 exhibited potent antimicrobial activity MIC (3.75 μg/mL) with minimal cytotoxicity toward epithelial cells, comparable to recent high-performing antimicrobial peptides (Mwangi et al., 2019; Spencer et al., 2018; Yang L. et al., 2024). Notably, F1/F3 selectively reduced intracellular rather than extracellular *A. baumannii* burdens in infected A549 cells. The apparent discrepancy between the strong antibacterial activity observed in MIC assays (3.75 μg/mL) and the limited reduction of extracellular bacteria in the host-cell infection model likely reflects differences in the experimental environments. MIC assays are performed in Mueller–Hinton broth under defined conditions, whereas the infection model involves a complex cellular milieu containing serum components and host-derived factors that may partially blind or attenuate peptide activity. Despite this, F1/F3 significantly reduced intracellular bacterial burden following gentamicin clearance of extracellular bacteria (from 6.4 log to 5.54 log). These findings suggest that F1/F3 may retain activity against intracellular bacterial reservoirs within host cells, a property reported for certain HDPs capable of accessing intracellular compartments. This capacity to reduce intracellular bacterial survival may contribute to the overall antibacterial efficacy of F1/F3 in cellular and *in vivo* infection contexts. This pattern suggests that F1/F3 possess cell-penetrating properties and act on intracellular bacterial reservoirs, similar to a small subset of host defense peptides capable of translocating across plasma membranes (Zhu et al., 2025). The ability to suppress intracellular bacteria is particularly important because intracellular persistence is a key determinant of chronic *A. baumannii* infection.

F1/F3 pretreatment of bacteria further reduced intracellular bacterial burden and alleviated epithelial cytotoxicity, as reflected by decreased LDH release (Nieto-Marín et al., 2025). The observation that F1/F3 pretreatment significantly reduced intracellular bacterial burden without affecting extracellular bacterial load suggests that the combination may impair bacterial invasion into host cells or reduce intracellular bacterial survival (Xiao et al., 2022). Furthermore, the differential effects on apoptosis—reduced early apoptosis but increased late apoptosis without altering total apoptosis—indicate that F1/F3 modulates the kinetics of apoptosis rather than simply suppressing overall cell death (Nieto-Marín et al., 2025). The peptides also attenuated infection-induced ROS accumulation, aligning with antioxidant and cytoprotective functions reported for LL-37 and other defense peptides in epithelial injury models (Di Bona et al., 2024; Gao et al., 2025; Ong and Logue, 2023; Ornatowski et al., 2020). Such a mechanism has been described for the human cathelicidin LL-37, which exerts cell-type-specific effects on apoptotic pathways, including the inhibition of neutrophil apoptosis while promoting apoptosis in other cell types (Simpson et al., 2011). By accelerating the apoptotic trajectory, F1/F3 may help preserve epithelial barrier integrity during acute infection (by reducing the accumulation of early apoptotic cells) while still allowing for the orderly clearance of damaged cells via efferocytosis. This efficient clearance of apoptotic cells is known to reprogram macrophages into a pro-repair phenotype through the secretion of anti-inflammatory mediators (such as TGF-*β*), metabolic reprogramming, and the production of growth factors (Li et al., 2026), consistent with the repair phenotype observed in F1/F3-treated tissues. Endocytosis-induced macrophage M2 polarization is a crucial step in inflammation resolution and tissue repair (Salina et al., 2022). These two mechanisms may work synergistically to facilitate tissue resolution and repair while minimizing tissue damage.

At the cytokine level, F1/F3 upregulated IL-6 without altering TNF-*α* production. The selective induction of IL-6 may reflect activation of classical (regenerative) rather than *trans*-signaling (pro-inflammatory) pathways (Stepp and Menko, 2021; Yu et al., 2025), thus supporting epithelial repair without amplifying harmful inflammation.

The *in vivo* murine skin-injury model further demonstrated the therapeutic potential of F1/F3. Topical F1/F3 treatment significantly reduced bacterial colonization, improved tissue architecture, and decreased inflammatory infiltration. Immune profiling revealed a coordinated reshaping of the cutaneous immune landscape. Macrophages increased in frequency and polarized from inflammatory M1 to reparative M2 phenotypes, consistent with wound-healing paradigms emphasizing macrophage-driven repair (Hoteit et al., 2023; Mao et al., 2025; Xu et al., 2021). The phenotypic shift observed in macrophage subsets following F1/F3 treatment was supported by complementary marker analyses. In the flow cytometry analysis, Ly6C gating was used as an initial approach to distinguish inflammatory (Ly6C^+^) from tissue-associated (Ly6C^−^) macrophage populations, a strategy commonly applied in murine infection and inflammation models (Stepp and Menko, 2021). To more specifically assess macrophage polarization, multiplex immunofluorescence staining was performed for CD86 (M1-associated marker) and CD206 (M2-associated marker). These analyses confirmed a decrease in CD86^+^ macrophages and a corresponding increase in CD206^+^ macrophages in F1/F3-treated tissues, supporting a shift from a pro-inflammatory toward a reparative macrophage phenotype. These complementary analyses support the conclusion that F1/F3 treatment promotes a macrophage phenotype associated with tissue repair and resolution of inflammation. Neutrophil infiltration, which is a major contributor to tissue-destructive inflammation, was substantially reduced, while dendritic cell recruitment increased, suggesting enhanced antigen presentation and priming of adaptive immune responses (He et al., 2024; Huete-Carrasco et al., 2025; Tiwary et al., 2025). Multiplex immunofluorescence confirmed these system-level shifts, illustrating a transition from a destructive to a reparative inflammatory milieu.

Cytokine profiling supported these cellular changes, showing upregulation of IL-12 and IFN-*γ* (Th1-skewing antibacterial responses), as well as IL-10 and TGF-*β* (anti-inflammatory and tissue-repair regulatory mediators) (Goudarzi et al., 2020; Ihara and Yamamoto, 2024; Moradi et al., 2020; Wang B. et al., 2021; Zhou et al., 2023). Elevated IL-6 and TGF-β further point toward activation of STAT3-mediated regenerative signaling (Li et al., 2023; Liu et al., 2023). The combination of increased dendritic cells and IL-12/IFN-γ activation suggests that F1/F3 may promote features of trained immunity, enabling more rapid and effective responses upon reinfection (Domínguez-Andrés et al., 2023; Ochando et al., 2023). Together, these findings reveal that F1/F3 establishes a tri-layered defense strategy involving direct antimicrobial activity, immune microenvironment reprogramming, and enhancement of tissue-repair pathways. This integrated host-directed mechanism expands the functional scope of caerin peptides and highlights their potential as therapeutic scaffolds for combating MDR infections.

Our findings align with and extend several recent studies from our group that characterized antibacterial and immune-modulatory attributes of caerin peptides in diverse biological contexts. Early work established that F1/F3 exhibit potent, broad-spectrum activity against both Gram-positive and Gram-negative pathogens, including MDR *A. baumannii* and MRSA, primarily through membrane-disruptive actions and without detectable resistance development after repeated passage (Chen et al., 2021). Building on this, caerin peptide formulations have demonstrated significant translational promise: a F3 antimicrobial gel reduced early bacterial infection around oral titanium implants in an animal model (You et al., 2026), and F3–coated titanium plates both inhibited bacterial growth and supported better implant healing in rabbit mandibles (Long et al., 2024). Similarly, degradable magnesium implants with F3–polycaprolactone coatings provided extended antibacterial activity while maintaining excellent biocompatibility *in vitro* (Liu et al., 2025). These studies collectively highlight the ability of caerin peptides to function as effective antibacterial agents in complex, clinically relevant environments. More recent work from our group in tumor models also showed that F1/F3 can modulate the immune microenvironment, enhancing macrophage infiltration and inflammatory responses to improve immunotherapeutic efficacy (Fu et al., 2024b; Xiao et al., 2022; Fu et al., 2024a). In contrast to the pro-inflammatory context of cancer, the present study reveals that F1/F3 can shape the immune response in infected tissue by promoting a reparative macrophage phenotype, reducing excessive neutrophil accumulation, and enhancing dendritic cell recruitment. Together, these data support a model in which caerin peptides act as context-dependent immune modulators that integrate direct antimicrobial activity with tailored regulation of host immunity and tissue repair, extending their potential applicability across infection, inflammation, and regenerative medicine.

While this study demonstrates the antimicrobial and immunomodulatory potential of the F1/F3 peptide combination, several limitations should be acknowledged. First, the experiments were conducted using a single *A. baumannii* strain, which may not fully represent the genetic and phenotypic diversity of MDR clinical isolates. Future studies should therefore evaluate the activity of F1/F3 against a broader panel of clinical strains from different geographic and resistance backgrounds to determine the generalizability of these findings. Second, although our results suggest that F1/F3 can access intracellular bacterial reservoirs and modulate macrophage polarization, the precise mechanisms underlying intracellular targeting and immune reprogramming remain to be elucidated. Further studies combining peptide tracking, host signaling analysis, and transcriptomic profiling will help clarify these pathways. Finally, a comprehensive evaluation of the pharmacokinetics, tissue distribution, and long-term safety of F1/F3 *in vivo* will be necessary to support its translational development as a therapeutic candidate. Notably, the relatively narrow therapeutic window suggested by the proximity of the IC₅₀ (7.876 μg/mL) and MIC (3.75 μg/mL) values (selectivity index ≈ 2.1) highlights the need for careful dose optimization in future translational development.

While our results provide functional evidence consistent with the intracellular antibacterial activity of F1/F3, the present study did not include dedicated peptide uptake or localization experiments. We therefore acknowledge that direct visualization of peptide internalization would further clarify the mechanism of action. Future studies will incorporate peptide tracking approaches, such as fluorescent labeling combined with time-resolved uptake analysis and subcellular colocalization with endosomal or lysosomal markers. These experiments will help define the intracellular trafficking routes of F1/F3 and determine the cellular compartments in which the peptides interact with intracellular bacterial reservoirs. Future studies should employ time-course infection assays and intracellular survival assays to distinguish whether F1/F3 pretreatment primarily affects bacterial entry into host cells or subsequent intracellular survival.

The translational potential of F1/F3 is supported by the broader clinical and translational experience with peptide-based anti-infective agents. Antimicrobial-peptide-based approaches have already been explored in wound repair, as exemplified by the clinical evaluation of LL-37 in hard-to-heal venous leg ulcers (Mahlapuu et al., 2021). In addition, AMP-derived candidates such as PL-5 (peceleganan) (Wei et al., 2023) and OMN6 (Shusterman Muratov et al., 2025) have progressed into clinical evaluation for skin wound infections and for hospital-acquired or ventilator-associated pneumonia caused by the *Acinetobacter baumannii* complex, respectively, underscoring the feasibility of peptide-based anti-infective strategies while also illustrating the challenges of clinical translation. In light of our findings that F1/F3 reduced bacterial colonization, improved tissue repair, and reshaped the local immune microenvironment, this peptide combination may have potential for further development as a topical therapeutic formulation for MDR *A. baumannii* wound infections. Future work should focus on optimizing delivery systems, defining pharmacokinetic and safety profiles, and validating efficacy in clinically relevant infection settings.

## 5 Conclusion

This study demonstrates that F1/F3 protects against *A. baumannii* infection through a dual mechanism involving both direct antibacterial activity and targeted modulation of the host immune microenvironment. F1/F3 effectively suppresses bacterial growth while reducing oxidative stress and epithelial injury, promoting macrophage polarization toward a reparative M2 phenotype, and enhancing dendritic cell recruitment and function. These coordinated actions facilitate efficient bacterial clearance together with accelerated tissue restoration. Collectively, our findings highlight F1/F3 not only as potent antimicrobial peptides but also as modulators of immune ecosystem dynamics, offering a conceptual and mechanistic foundation for developing next-generation host-directed therapies against MDR infections.

## Data Availability

The original contributions presented in the study are included in the article/Supplementary material, further inquiries can be directed to the corresponding author/s.
