# Supplementary material for: Caerin 1.1/1.9 peptides control Acinetobacter baumannii infection through combined antibacterial and host-directed immunomodulatory mechanisms
Source: Front Microbiol. 2026 May 14;17:1777814. doi: 10.3389/fmicb.2026.1777814 (PMC13226875; doi:10.3389/fmicb.2026.1777814)
Supplement: Supplementary file 1 [file Data_Sheet_1.pdf]

Supplementary Material

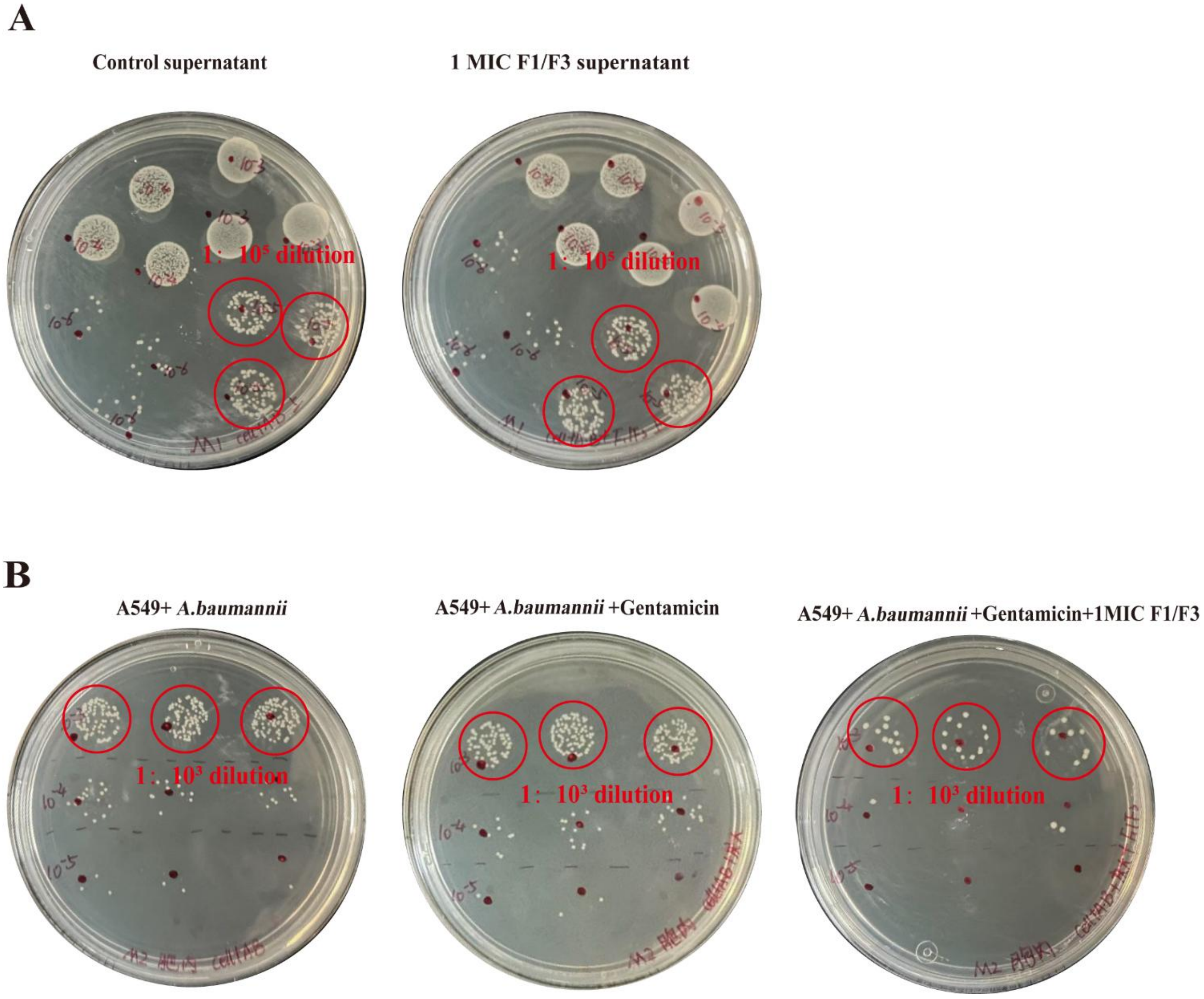

Supplementary Figure 1.A. Representative spot-plating images corresponding to panel **Fig.2A.B.**Representative spot-plating images corresponding to panel **Fig.2B.**

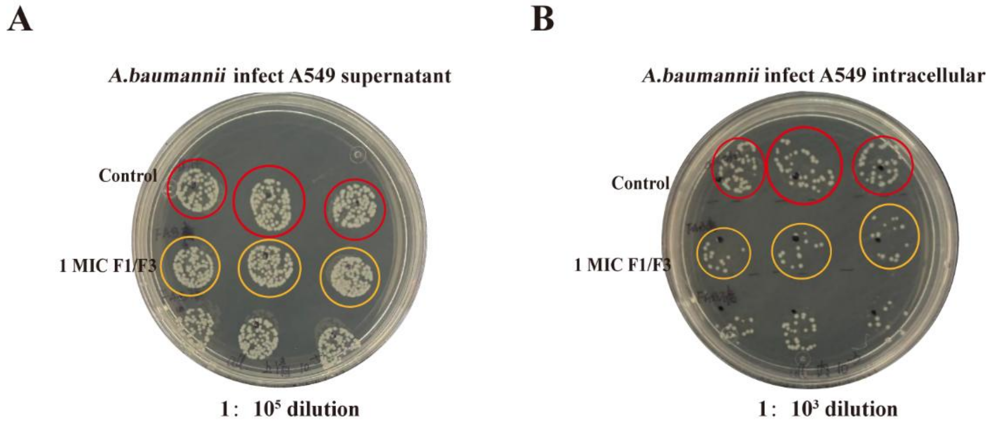

Supplementary Figure 2.A. Representative spot-plating images corresponding to panel **Fig.3B.** **B.**Representative spot-plating images corresponding to panel **Fig.3C.**

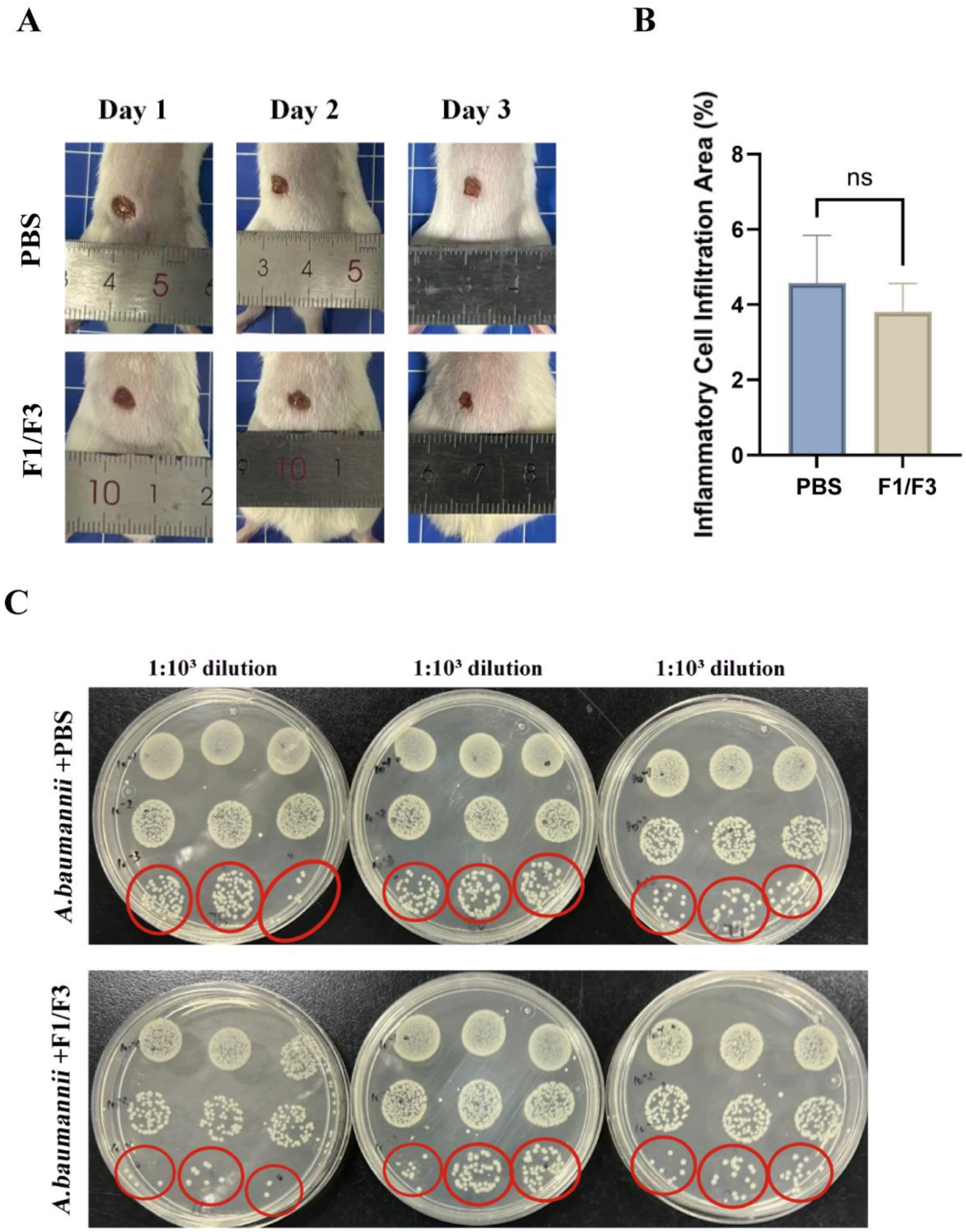

Supplementary Figure 3. A: Bacteria (1 × 10^8 CFU) were inoculated into each wound for 1 day, followed by treatment with F1/F3 (10 μg/ml) or PBS l for 2 days (n=3mice per group). Wound healing was then assessed. **B:** Mouse skin tissue sections from the PBS group and the F1/F3 group were stained with hematoxylin and eosin. The percentage of inflammatory cell infiltration area was quantitatively analyzed using ImageJ software.**C.**Representative spot-plating images corresponding to panel **Fig.4B.** Results are expressed as mean ± SEM. ns indicates no statistically significant difference. Statistical analysis was performed using an unpaired t-test.

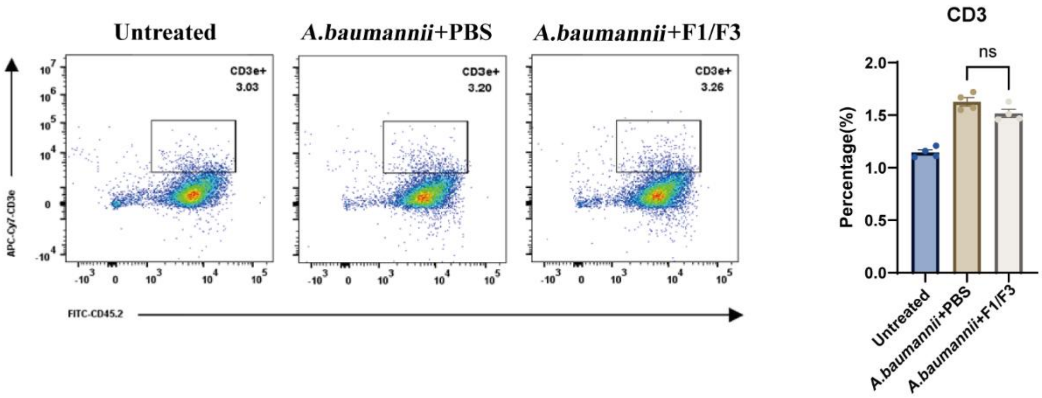

Supplementary Figure 4.T cell（*CD45$^+$CD3$^+$*）.Results are expressed as mean ± SEM. ns indicates no statistically significant difference. Statistical analysis was performed using unpaired t-tests.

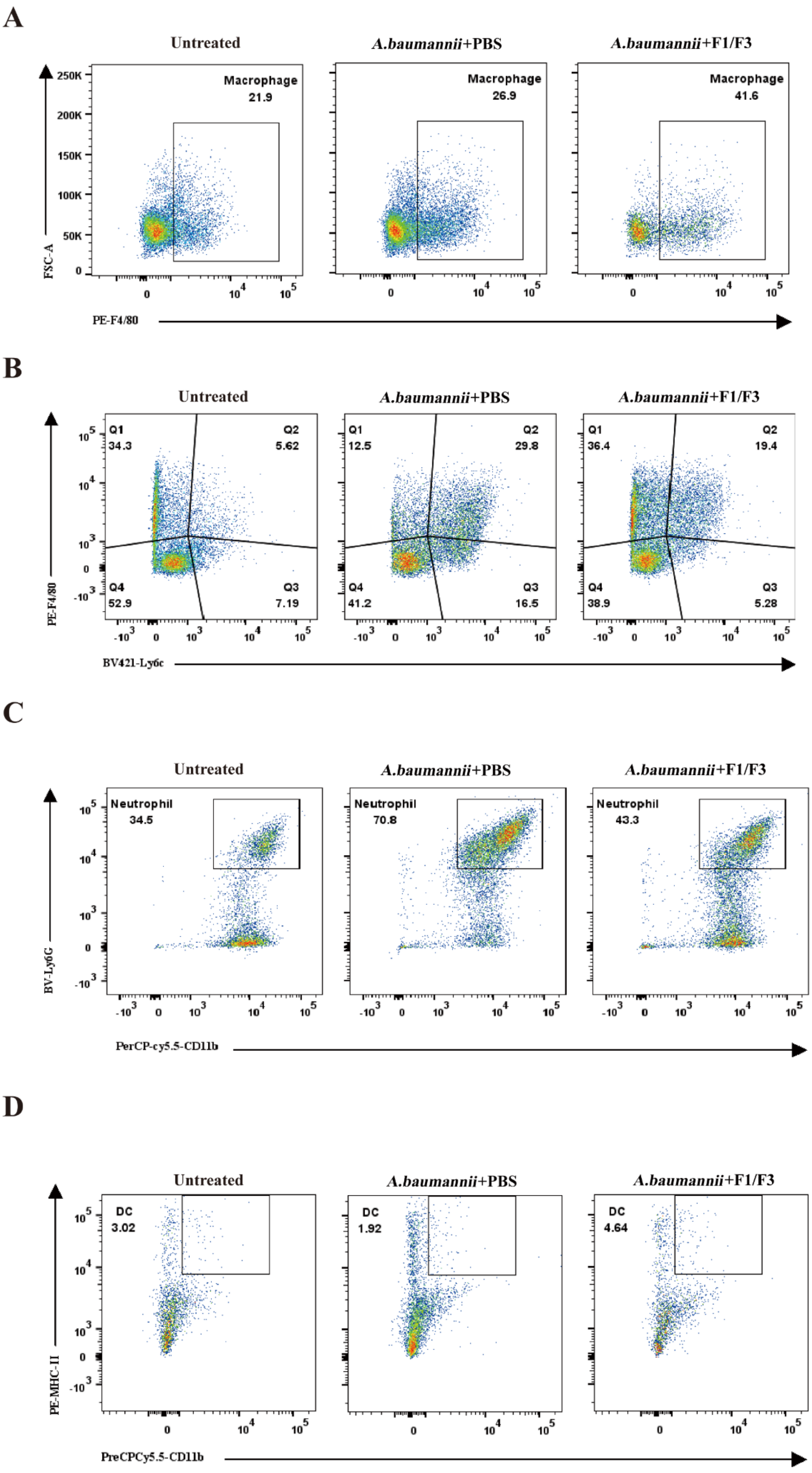
A
Untreated
A.baumannii+PBS
A.baumannii+F1/F3
Macrophage
21.9
Macrophage
26.9
Macrophage
41.6
FSC-A
PE-F4/80
B
Untreated
A.baumannii+PBS
A.baumannii+F1/F3
Q1 34.3
Q2 5.62
Q4 52.9
Q3 7.19
Q1 12.5
Q2 29.8
Q4 41.2
Q3 16.5
Q1 36.4
Q2 19.4
Q4 38.9
Q3 5.28
PE-F4/80
BV421-Ly6c
C
Untreated
A.baumannii+PBS
A.baumannii+F1/F3
Neutrophil
34.5
Neutrophil
70.8
Neutrophil
43.3
BV-Ly6G
PerCP-cy5.5-CD11b
D
Untreated
A.baumannii+PBS
A.baumannii+F1/F3
DC
3.02
DC
1.92
DC
4.64
PE-MHC-II
PreCPCy5.5-CD11b

Supplementary Figure 5.Gating proportion plot of neutrophils/dendritic cells/macrophages in flow cytometry analysis of skin tissue.A. Representative flow cytometry plot of macrophages from **Fig.5A**. The plot shows the percentage of gated cells.**B**.Representative flow cytometry plot of *M1-like and M2-like* from **Fig.5B**. The plot shows the percentage of gated cell.**C**.Representative flow cytometry plot of neutrophil from **Fig.5C**. The plot shows the percentage of gated cell. **D**.Representative flow cytometry plot of dendritic cell from **Fig.5D**. The plot shows the percentage of gated cell.

A

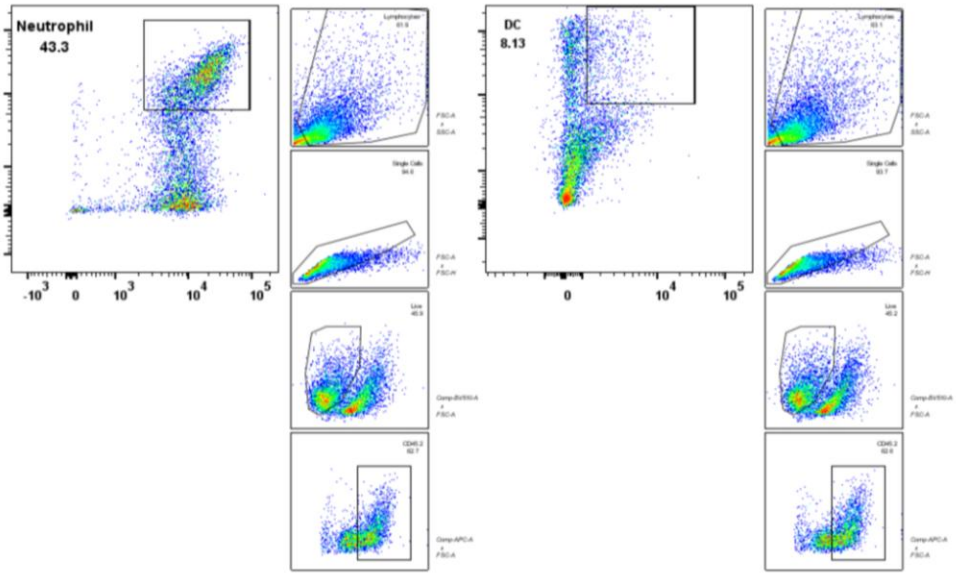

B

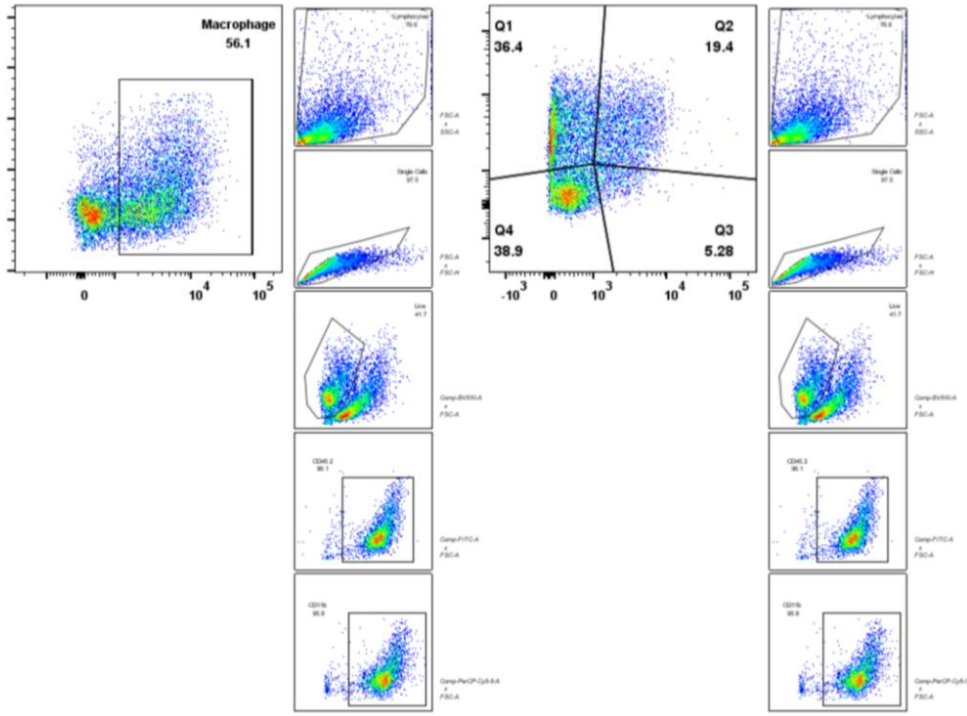

Supplementary Figure 6.***Gating trategy for Flow Cytometric Analysis of Neutrophil/DC/ Macrophage cells in skin tissue.***

A:*Neutrophil （$CD45^{+}CD11b^{+}Ly6G^{+}$）； Dendritic cell（$CD45.2^{+}CD11c^{+}MHCII^{+}$）；*
B:*Macrophage（$CD45^{+}CD11b^{+}F4/80^{+}$）, M1-like (Q2, $CD45^{+}CD11b^{+}F4/80^{+}Ly\ 6C^{+}$) and M2-like (Q1,$CD45^{+}CD11b^{+}F4/80^{+}Ly\ 6C^{-}$)*
